# Obituary

**Published:** 1902-05-15

**Authors:** 


### OBITUARY.

### DR. HENRY BLISS NOBLE.

Dr. Henry Bliss Noble, a prominent and highly esteemed practitioner of Washington, D. C., died suddenly from heart disease, while riding in a street car on the morning of March 5, 1902.

Born May 20, 1832, in Blandford, Mass., Dr. Noble began the study of dentistry with his brother, Dr. Lester Noble, March 2, 1857, and after graduating at the Baltimore College of Dental Surgery (March 3, 1859), immediately entered upon the practice of dentistry in Washington, D. C., continuing in active practice until the date of his decease. He was a member of the District of Columbia Dental Society, having been twice elected its president, and a member of the District of Columbia Board of Dental Examiners from the date of its organization until his death. He was also a member of the National Dental Association. He was special lecturer at the Baltimore College of Dental Surgery, also at the Dental Department of Columbia University.

In 1864 Dr. Noble was married to Miss Henrietta Clitch, of Washington, D. C. Three daughters survive him, Miss Irene Noble, Mrs. Clagett, of Linden, Md., and Mrs. Marshall, of Pittsburg, Pa. Dr. Noble's wife died several years ago. A son, who became a practicing dentist, also died several years ago.

Dr. Noble was very successful in dental practice, being regarded as a skillful operator in all branches of dentistry and especially so in the correction of dental irregularities. He was devoted to his profession, always enthusiastic and energetic in his efforts to promote its advancement. He was actively interested in the work of his local dental society, rarely missed a meeting, and greatly enjoyed discussing the various subjects presented. Notwithstanding his age he was always young in his devotion to his calling, and considered no sacrifice of time and labor too great for it. He was very loyal to his friends, was beloved by them and generally respected as a citizen; faithful in his devotion to his church, and benevolent and kindly, his attitude was one of malice toward none and charity for all.

Faithful, honorable, and upright in all of his vital relations, his death causes a loss which will be severely felt by the dental profession and by the community in which his active life was spent.—*Cosmos*.

